# Supplementary material for: Maraviroc Prevents HCC Development by Suppressing Macrophages and the Liver Progenitor Cell Response in a Murine Chronic Liver Disease Model
Source: Cancers (Basel). 2021 Sep 30;13(19):4935. doi: 10.3390/cancers13194935 (PMC8508380; doi:10.3390/cancers13194935)
Supplement: Supplementary file 1 [file cancers-13-04935-s001.zip › cancers-1321045-supplementary.pdf]

cancers

MDPI

Supplementary Materials: Maraviroc Prevents HCC Development by Suppressing Macrophages and the Liver Progenitor Cell Response in a Murine Chronic Liver Disease Model

Adam M. Passman, Robyn P. Strauss, Sarah B. McSpadden, Megan Finch-Edmondson, Neil Andrewartha, Ken H. Woo, Luke A. Diepeveen, Weihao Zhao, Joaquín Fernández-Irigoyen, Enrique Santamaría, Laura Medina-Ruiz, Martyna Szpakowska, Andy Chevigné, Hyerin Park, Rodrigo Carlessi, Janina E. E. Tirnitz-Parker, José R. Blanco, Roslyn London, Bernard A. Callus, Caryn L. Elsegood, Murray V. Baker, Alfredo Martínez, George C.T. Yeoh and Laura Ochoa-Callejero

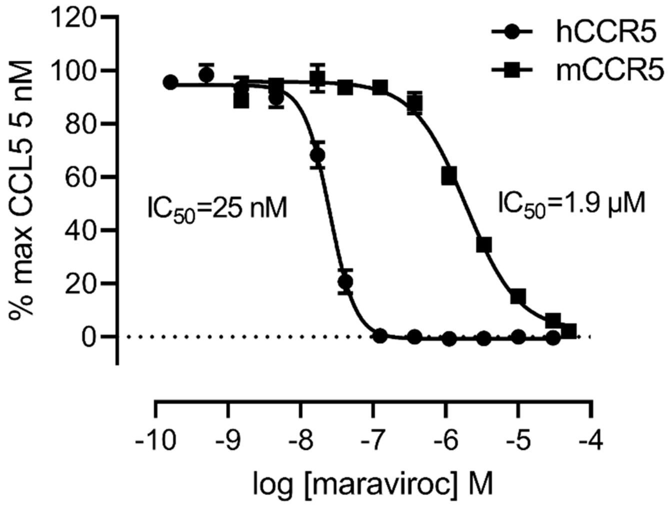

Figure S1. Comparative inhibition of human and mouse CCR5 by MVC. Inhibition of CCL5-induced (5 nM) human (circle) or mouse (square) CCR5 activation by increasing concentrations of Maraviroc was monitored in HEK293T cells using β-arrestin-1 recruitment assay based on Nanoluciferase complementation (NanoBiT). Concentration-response curves were fitted to the four-parameter Hill equation using an iterative, least-squares method (GraphPad Prism version 8.0.1). All curves were fitted to data points generated from the mean of five independent experiments.

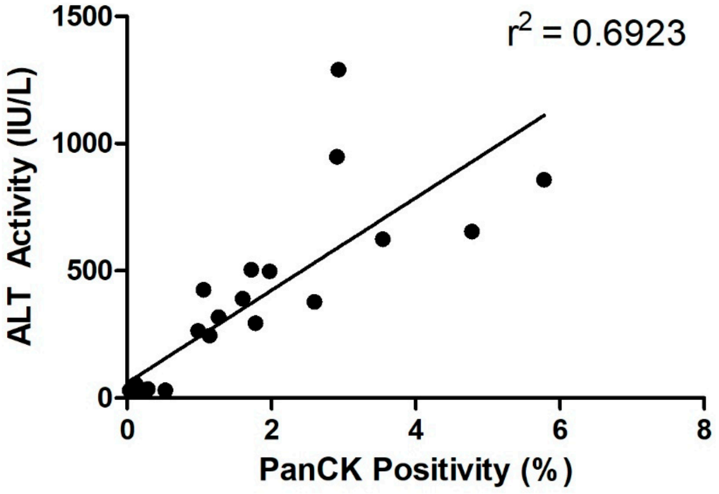

Figure S2. Correlation of PanCK staining with ALT. A correlation between the serum alanine aminotransferase activity levels of 16-wk control, MVC, CDE, and CDE + MVC mice and their corresponding liver PanCK positivity values (*n* = 26).

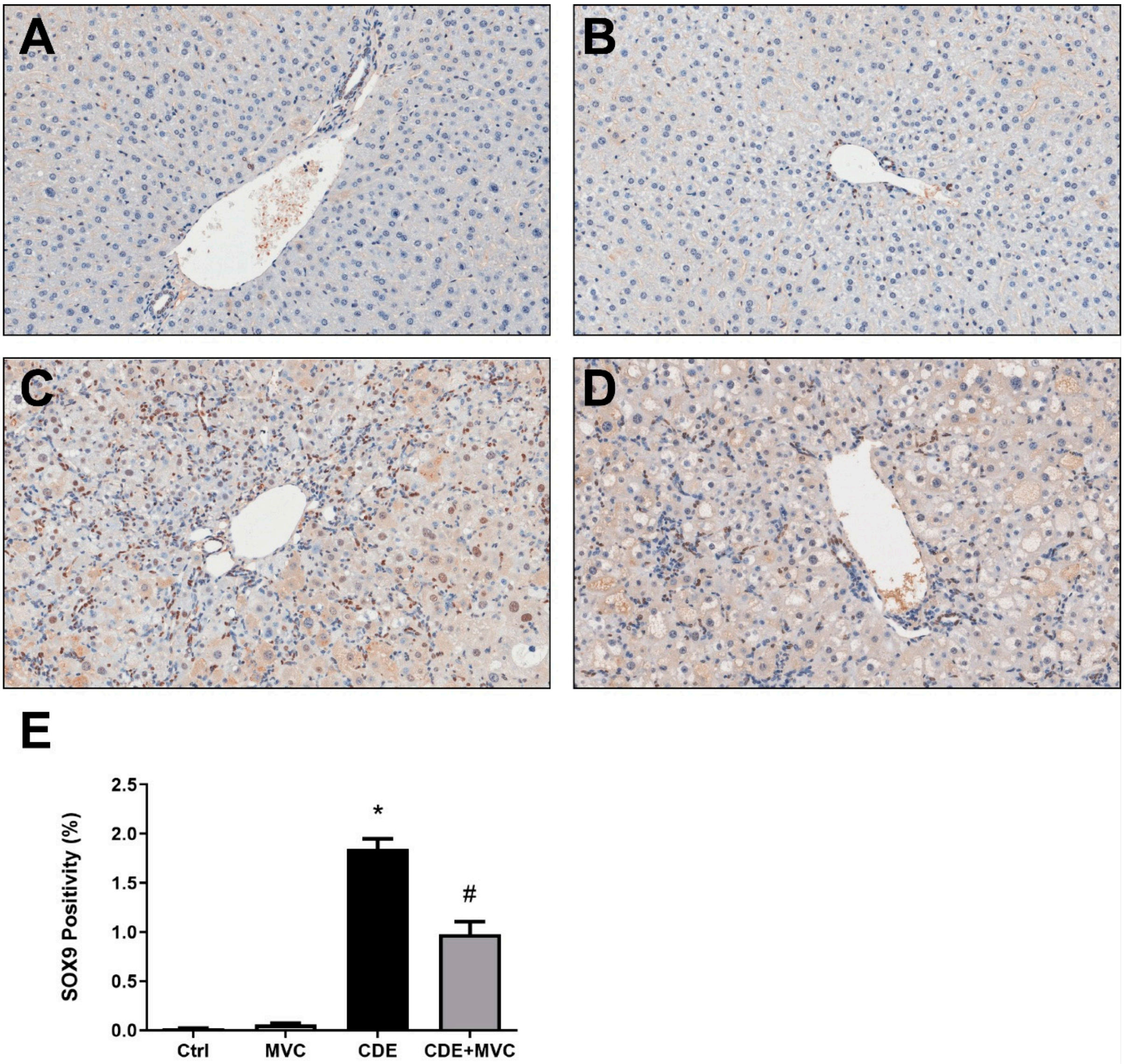

Figure S3. The number of SOX9 positive cells is reduced by MVC in CDE liver. (**A–D**) Representative images of histological sections stained with SOX9 antibody, from 16-wk control (**A**), MVC (**B**), CDE (**C**), and CDE + MVC (**D**) animal groups. (**E**) Quantitation of Sox9 staining by pixel positivity. Bars represent means + SEM for n = 4, 3, 4 and 4 in the control, MVC, CDE, and CDE + MVC groups respectively. * $p < 0.05$ compared to control. # $p < 0.05$ compared to CDE group.

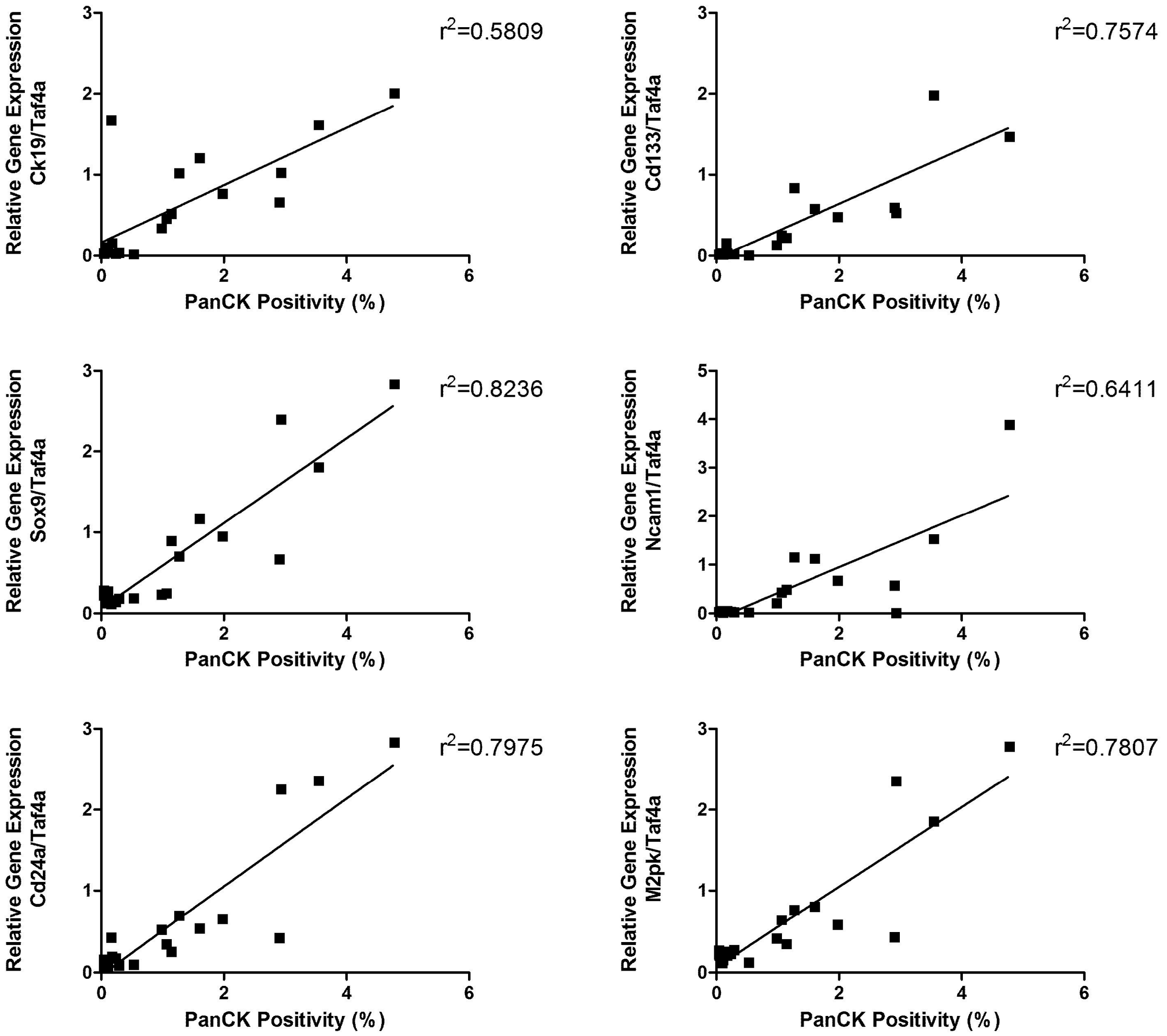

Figure S4. Correlation of PanCK staining with LPC gene expression. A correlation between the normalised expression values of six LPC genes in livers from 16-wk control, MVC, CDE, and CDE + MVC mice, and their corresponding PanCK positivity values (*n* = 20).

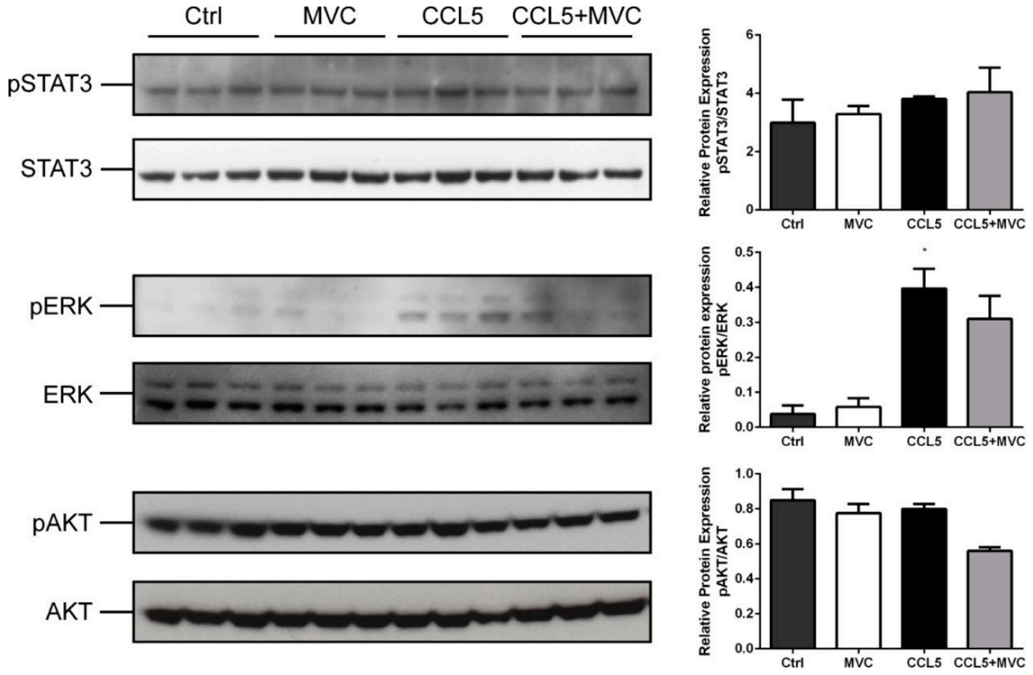

Figure S5: pSTAT3 and pAKT expression is unaffected by MVC in LPCs. Protein lysates for LPCs +/- MVC and/or +/- CCL5 were immunoblotted and labeled for phosphorylated STAT3, pERK, pAKT, and their loading controls; total STAT3, ERK, and AKT. Right; The abundance of each phosphorylated protein relative to its control was calculated through densitometry performed on the blot images. Bars represent means + SEM for $n = 3$ in each test group. * $p < 0.05$ compared to control.

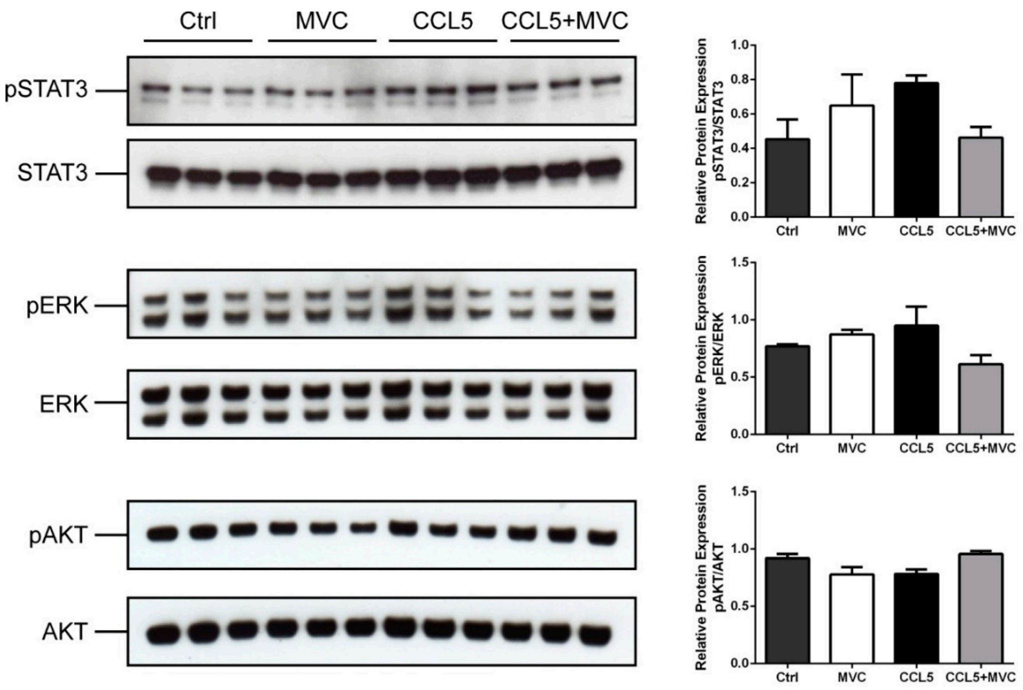

Figure S6. pSTAT3 and pAKT expression is unaffected by MVC in BMMOs. Protein lysates for BMMOs +/- MVC and/or +/- CCL5 were immunoblotted and labeled for phosphorylated STAT3, pERK, pAKT, and their loading controls; total STAT3, ERK, and AKT. Right; The abundance of each phosphorylated protein relative to its control was calculated through densitometry performed on the blot images. Bars represent means + SEM for n = 3 in each test group.

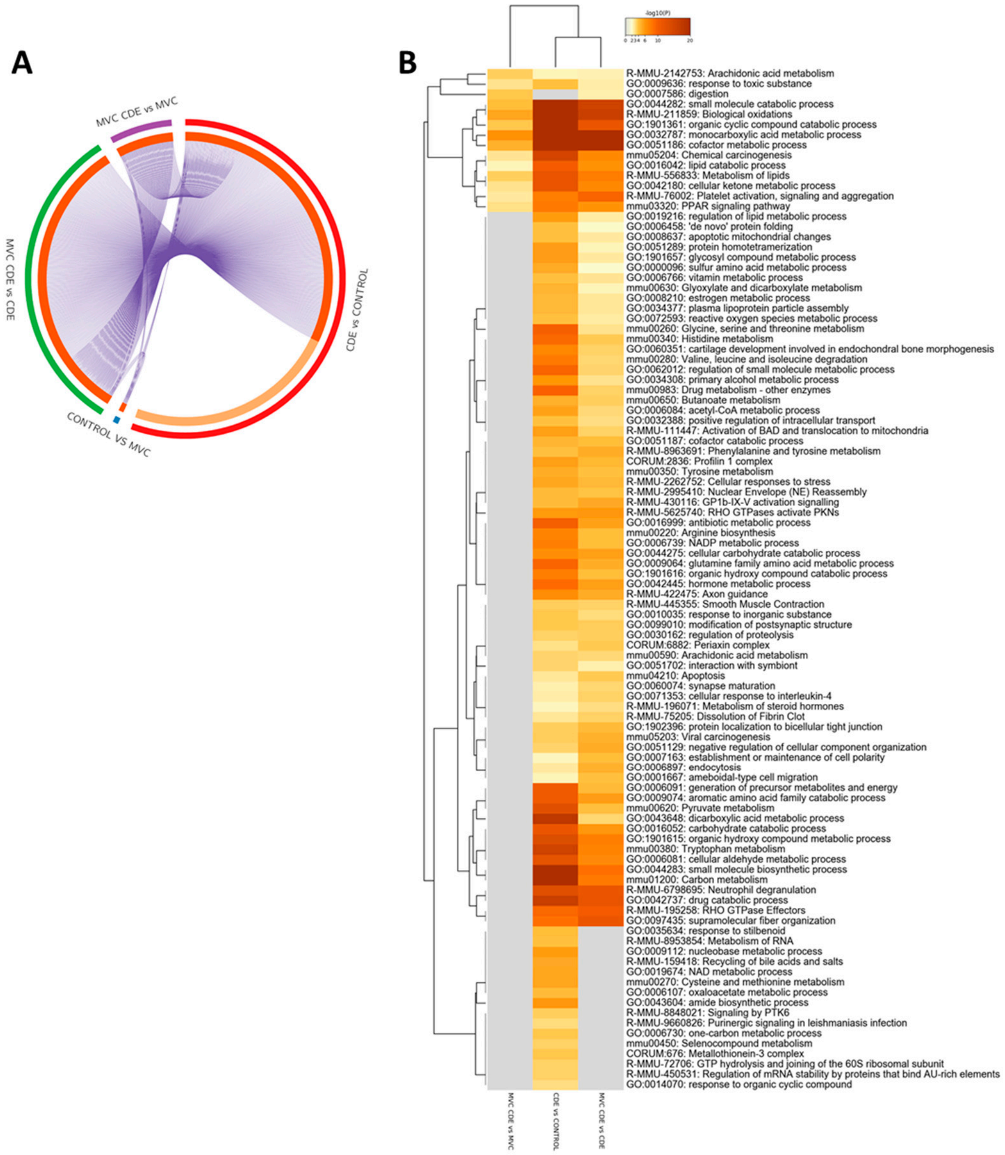

Figure S7: MVC attenuates CDE-induced proteome changes in the liver. Proteomic analysis was performed using an isobaric labelling approach (iTRAQ) (Unwin RD, Griffiths JR, Whetton AD.Nat Protocols. 2010). (**A**) Circos plots showing overlap of deregulated proteome between CDE and MVC treatments. Outside arc color identifies each comparative analysis by color. Inside arc represents the modulated proteome in each case; dark orange bars represent proteins shared between datasets and light orange bars represent non-overlapping proteins. (**B**) Dendrogram showing enriched ontology clusters across MVC, CDE and MVC + CDE-induced differential proteomes generated by Metascape [71]. The heat map cells are coloured by their *p*-values; grey cells indicate the lack of enrichment for that term in the corresponding biological condition. Due to the minimal changes observed between control and MVC conditions, this comparative analysis is not reflected in the functional clustering

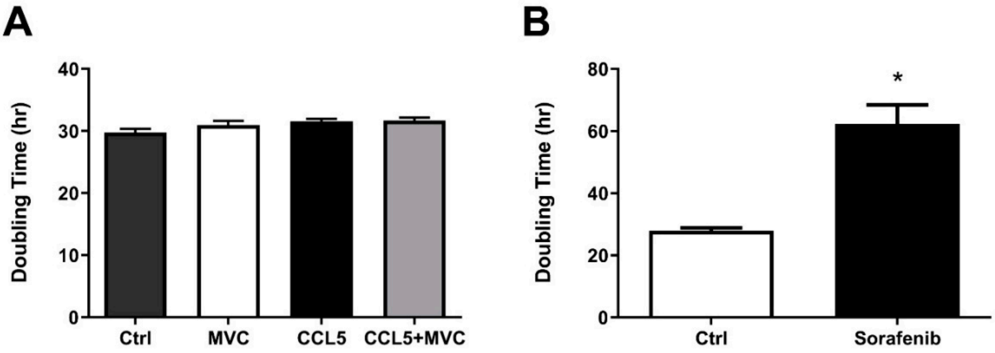

Figure S8. No effect of CCL5 and/or MVC on growth of LPC lines. (**A**) The proliferation doubling times of BMOL LPCs under regular growth conditions (Ctrl) or after treatment with 1 μM MVC and/or 50 ng/mL CCL5, as measured using the Cellavista apparatus. Bars represent means + SEM for $n = 8$. (**B**) Proliferation of BMOL LPCs during growth in regular medium or after treatment with 10 μM Sorafenib. Bars represent means + SEM for $n = 3$. * $p < 0.05$ compared to control.

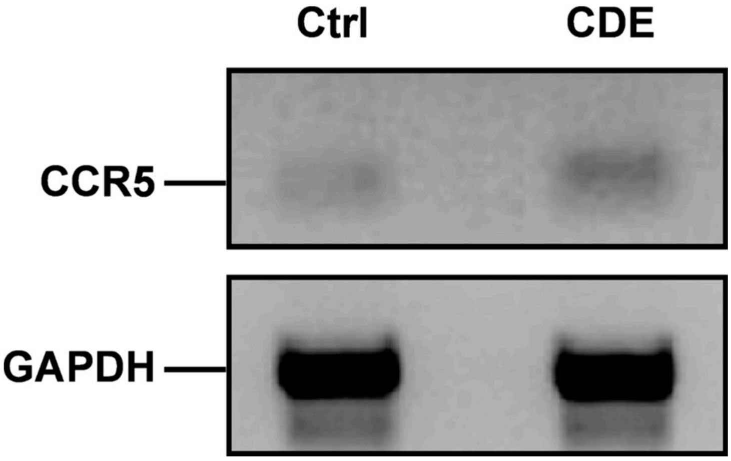

Figure S9. *Ccr5* expression is increased in CDE liver. RT-PCR was performed to amplify *Ccr5* and the *Gapdh* loading control using cDNA from control and CDE chow for 3 weeks. The *Ccr5* and *Gapdh* primers amplify 68 and 437 bp amplicons respectively.

Western Blots of Figure 3.

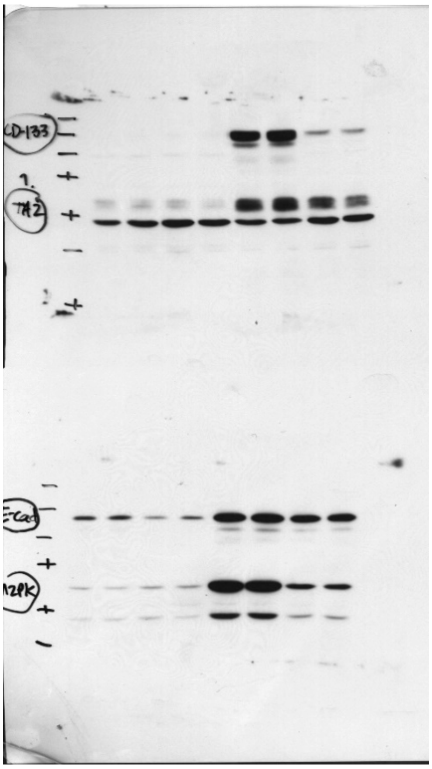

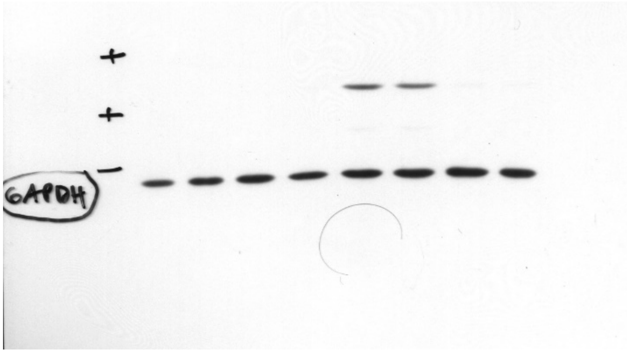

Western Blots of Figure 4.

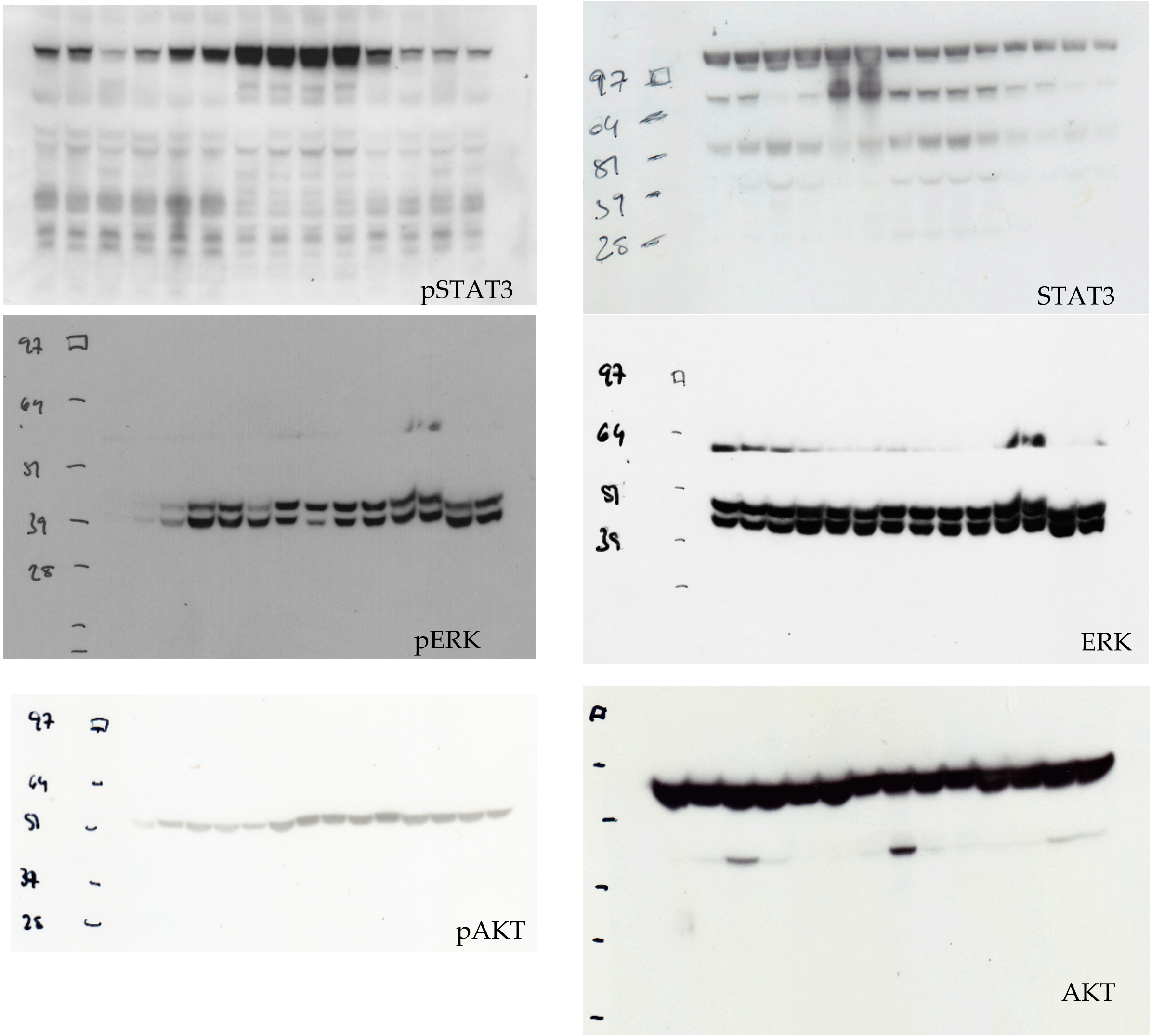

Western Blots of Figure 8.

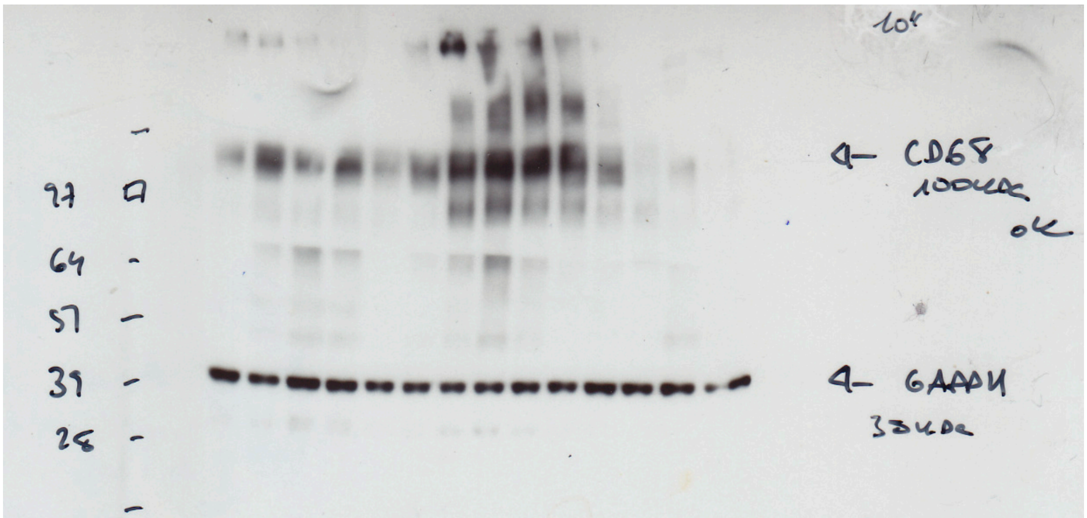

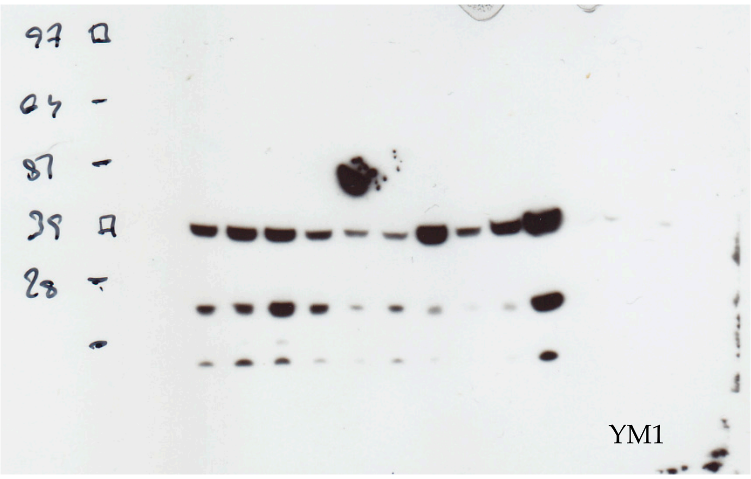

Western Blots of Figure S5.

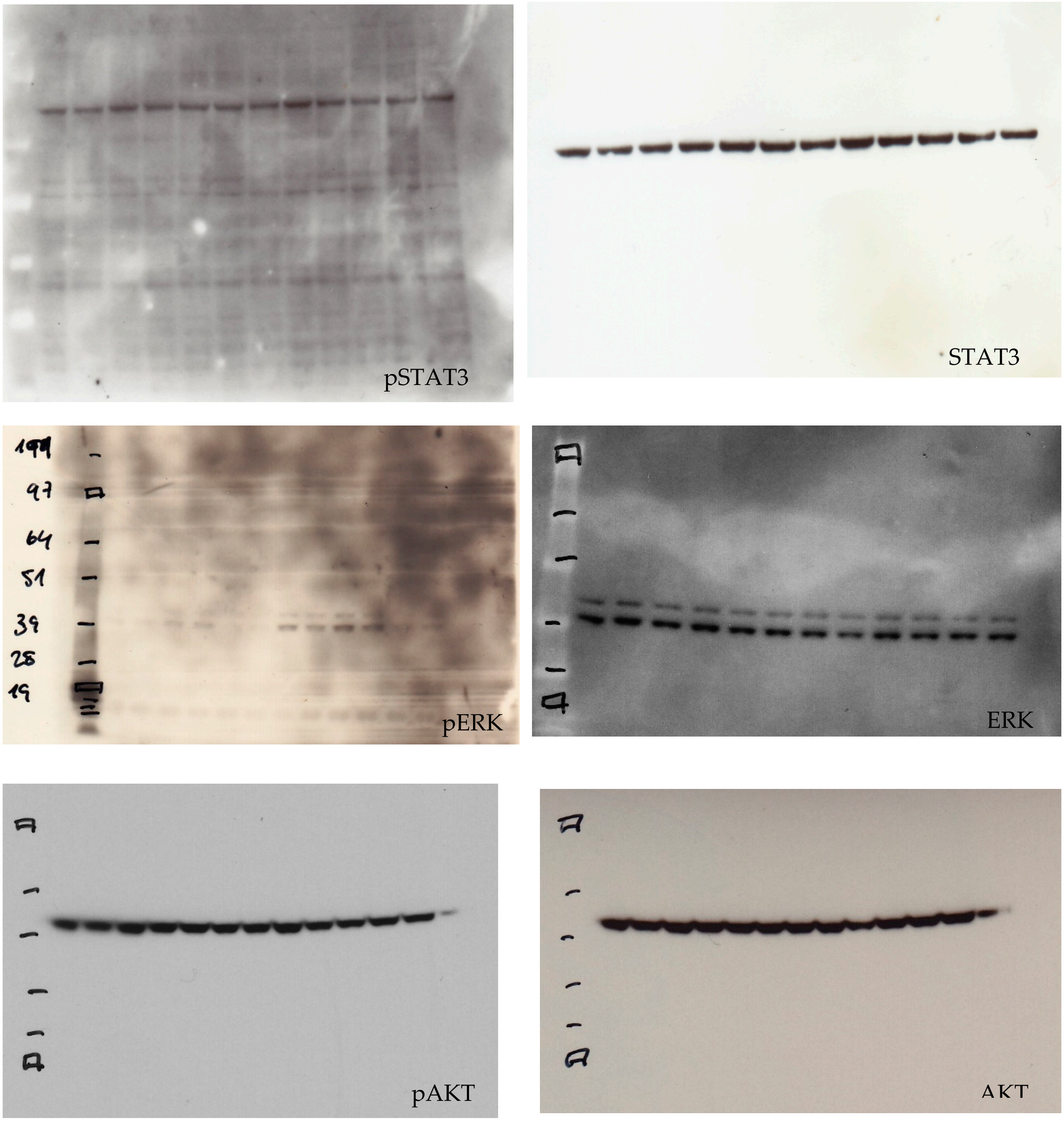

Western Blots of Figure S6.

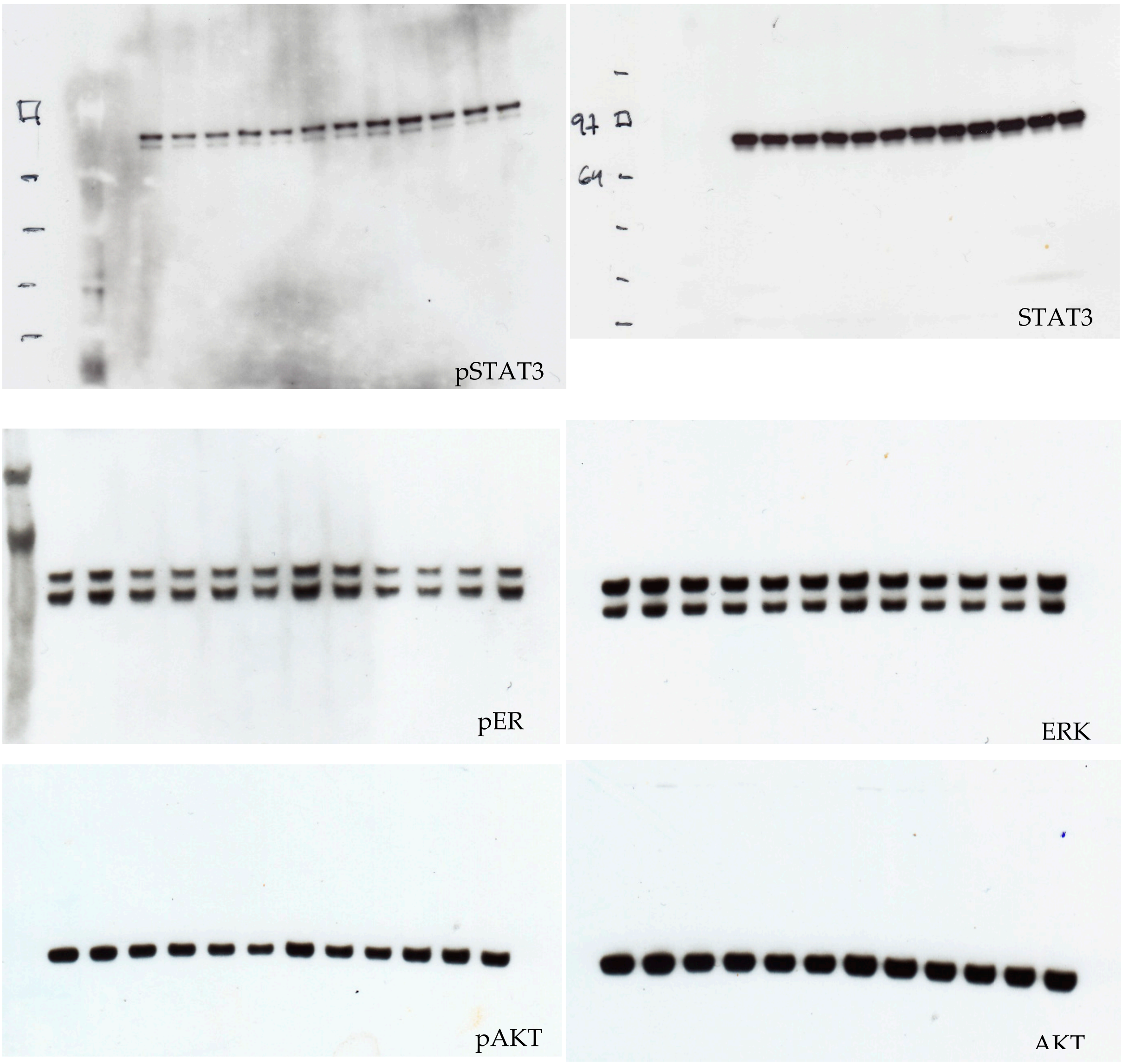

Figure S10. Full Western Blots Images.

Table S1. inForm settings.

Name of algorithm	Setting Category	Settings
CD45 algorithm	Images analysed	CD45 stained sections (IHC)
	Configuration settings	Segment images > trainable tissue segmentation<br>Find features > cell segmentation<br>Score > Score
	Image preparation settings	Image format > RGB<br>Sample resolution > Brightfield<br>Convert to optical density > select white<br>Spectral library > Brightfield<br>Spectra for unmixing > blue hematox, purple hematox, DAB
	Tissue segmentation settings	Tissue categories > CD45, parenchyma<br>Components for training > blue hematox, purple hematox, DAB<br>Pattern scale > small<br>Train tissue segmenter > 99.6%<br>Segmentation resolution > medium<br>Minimum segment size > 500 pixel
	Cell segmentation settings	Compartments to segment > nuclei<br>Nuclei segmentation<br>> Tissue category > all categories<br>> approach > object based<br>> signal scaling > auto scale<br>> Primary > blue hematox > min signal 0.01<br>> size range<br>> min size 350 pixels<br>> max sixe 10000 pixels<br>> clean-up<br>> fill holes<br>> refine splitting<br>> roundness min circularity 0.30
	Scoring setting	Tissue category > CD45<br>Scoring > Positivity (2-bin)<br>Compartment > Nuclei<br>Component > DAB<br>Threshold max > 0.80<br>Positivity threshold > 0.064
F4/80 algorithm	Images analysed	CD45 stained sections (IHC)
	Configuration settings	Segment images > trainable tissue segmentation<br>Find features > cell segmentation<br>Score > Score
	Image preparation settings	Image format > RGB<br>Sample resolution > Brightfield<br>Convert to optical density > select white<br>Spectral library > Brightfield<br>Spectra for unmixing > blue hematox, DAB

	Tissue segmentation settings	Tissue categories > F480, parenchyma, white<br>Components for training > blue hematox, DAB<br>Pattern scale > small<br>Train tissue segmenter > 85%<br>Segmentation resolution > fine
	Cell segmentation settings	Compartments to segment > nuclei<br>Nuclei segmentation<br>> Tissue category > all categories<br>> approach > object based<br>> signal scaling > auto scale<br>> Primary > blue hematox > min signal 0.01<br>> size range<br>> min size 300 pixels<br>> max sixe 5000 pixels<br>> clean-up<br>> fill holes<br>> max hole size 250 pixels<br>> refine splitting<br>> roundness min circularity 0.28
	Scoring setting	Tissue category > F480<br>Scoring > Positivity (2-bin)<br>Compartment > Nuclei<br>Component > DAB<br>Threshold max > 0.40<br>Positivity threshold > 0.058

Table S2. inform analysis workflow.

Process	Steps
Create Algorithm	1. Load 10–15 images that includes a representative range<br>Includes samples from all groups<br>Includes samples that show high and low amount PCK^{+ve} cells<br>Includes samples that have low and high background levels<br>2. Set parameters (given in supplementary Table S1)<br>3. Set training regions and processing regions (given in supplementary Table S1)<br>4. Train tissue segmenter. Ensure accuracy rate is above 80%<br>5. Save algorithm
Verify Algorithm	1. In photoshop, outline and crop individual PCK^{+ve} cells/ and also outline and crop cells/areas that do not show this feature (e.g. normal parenchyma). At least 50 cells/areas should be outlined and saved as TIFs. Note by, these cells/areas should be different than images chosen for creation of algorithm<br>2. Upload TIFs into inForm, run algorithm and analyse output. Ensure algorithm can accurately distinguish positive from negative features

Table S3. Primers used for qRT-PCR.

Name	Primer
Sox9	Sense: 5'-gaagctggcagaccagtacc-3'<br>Antisense: 5'-ggtctcttctcgctctcgttc-3'
Ncam	Sense: 5'-gtggtatgatgccaaagaagc-3'<br>Antisense: 5'-ccgagtacctcgtctcaggt-3'
CK19	Sense: 5'-gctggcctacctgaagaaga-3'<br>Antisense: 5'-atccacctccacactgacc-3'
CD133	Sense: 5'-gcccaagctggaagaatatg-3'<br>Antisense: 5'-cagcagaaagcagacaatcaa-3'
M2PK	Sense: 5'-aagggggactaccctctgg-3'<br>Antisense: 5'-cctcgaatagctgcaagtgg-3′
CD24a	Sense: 5'-cttctggcactgctcctacc-3'<br>Antisense: 5'-tggtggtagcgttacttgga-3'
Taf4a	Sense: 5'-ccacagcagatccaactgaa-3'<br>Antisense: 5'-ggtaacacggtgggtttcac-3'
Ccr5	Sense: 5'-cgaaaacacatggtcaaacg-3'<br>Antisense: 5'-ttcctactcccaagctgcat-3'
Gapdh	Sense: 5'-catgttccagtatgactccactc-3'<br>Antisense: 5'-ggcctcaccccatttgatgt-3'

Annealing temperature of all reactions was 60 ºC. Taf4a or Gapdh were used as housekeeping genes.

References

71. Zhou Y.; Zhou B.; Pache, L.; Chang; M.; Hadj Khodabakhshi, A.; Tanaseichuk, O.; Benner, C.; Chanda, S.K. Metascape provides a biologist-oriented resource for the analysis of systems-level datasets. *Nat. Commun.* **2019**, *10*, 1–10.
